# Cross-hemispheric recruitment during action planning with increasing task demand

**DOI:** 10.1038/s41598-023-41926-4

**Published:** 2023-09-16

**Authors:** Sonja Schach, Daniel Alexander Braun, Axel Lindner

**Affiliations:** 1 Institute of Neural Information Processing, University of Ulm, Ulm, Germany; 2 Tübingen Center for Mental Health, Department of Psychiatry and Psychotherapy, University of Tübingen, Tübingen, Germany; 3 Centre of Neurology, Division of Neuropsychology, Hertie-Institute for Clinical Brain Research, University of Tübingen, Tübingen, Germany

**Keywords:** Cognitive neuroscience, Motor control, Sensorimotor processing

## Abstract

The recruitment of cross-hemispheric counterparts of lateralized prefrontal brain regions with increasing processing demand is thought to increase memory performance despite cognitive aging, but was recently reported to be present also in young adults working at their capacity limit. Here we ask if cross-hemispheric recruitment is a general strategy of the adult brain in that executive task demand would modulate bilateral activation beyond prefrontal cortex and across cognitive tasks. We analyzed data sets from two fMRI experiments investigating retrospective working memory maintenance and prospective action planning. We confirmed a cross-hemispheric recruitment of prefrontal cortex across tasks and experiments. Changes in lateralization due to planning further surfaced in the cerebellum, dorsal premotor and posterior parietal cortex. Parietal cortex thereby exhibited cross-hemispheric recruitment also during spatial but not verbal working memory maintenance. Our results confirm a domain-general role of prefrontal cortex in cross-hemispheric recruitment. They further suggest that other task-specific brain regions also recruit their idling cross-hemispheric counterparts to relocate executive processing power.

## Introduction

Cognitive processes often display a marked left-right asymmetry in terms of their hemispheric representation within the brains of both humans and animals (e.g. see^1^). The earliest and most prominent examples in humans perhaps refer to the attribution of speech deficits to the left hemisphere^2–4^ and of attentional deficits to the right hemisphere^5^. This distinction has later been confirmed also by correlative approaches using functional imaging (e.g. see^6,7^). Functional lateralization is accompanied by hemispheric differences in brain anatomy (e.g. see^8^) and in network characteristics (e.g. see^6,9^). Such hemispheric specializations may - in addition to the anatomical segregation of cognitive functions within a hemisphere - increase our brain’s capacity for carrying out different cognitive operations simultaneously. Indeed, hemispheric specialization can improve sensory discrimination abilities as well as cognitive and motor processing (see^10,11^ for review); and it does so, supposedly, by freeing attentional capacity for the simultaneous processing of differently lateralized functions, and by avoiding redundancy in information processing^8,10,12–14^. Yet, this comes at the expense of greater vulnerability of lateralized systems due to pathology (e.g. see^10^).

Here we address what happens when the processing demands for any individual lateralized function approach its capacity limit. As we will detail below, earlier studies do suggest that in such situations lateralized brain regions that are related to this function would co-recruit their “idling” counterparts in the other hemisphere. Accordingly, the degree of hemispheric lateralization of these brain regions would decrease with increasing processing demands. In other words, when working at one’s capacity limit bilaterality, rather than lateralization would warrant high performance.

The notion that bilateral activation during high task demand would extend the capacity of otherwise lateralized brain processes was proposed, for instance, based on imaging experiments within the research field of cognitive aging. During verbal and spatial working memory (WM) tasks, for which processing capacity shows an age-related decline^15^, Reuter-Lorenz and colleagues^16^ observed more bilateral brain activity in frontal brain areas in older as compared to younger subjects. Around the same time, similar findings were revealed also by other research groups (e.g.^17,18^). For instance, Cabeza et al. ^18^, observed a reduction of hemispheric asymmetry in anterior prefrontal cortex (aPFC) in a group of high performing elderly subjects, an effect which these authors termed HAROLD (hemispheric asymmetry reduction in older adults), accordingly. Importantly, in their study this HAROLD effect was due to an increase of activity in the idling hemisphere in well performing subjects only, while bilateral activation in prefrontal cortex was missing in those elderly subjects, who could not successfully manage the increasing WM demand. In the meantime, several studies confirmed that increases in efficiency of mnemonic processing is associated with cross-hemispheric recruitment in prefrontal cortex (^19–21^, but see^22^ for an opposing result). Clearly, the successful compensation of age-related decline through such cross-hemispheric recruitment would still depend on the integrity of frontal circuits in individuals^23^.

**Figure 1:**
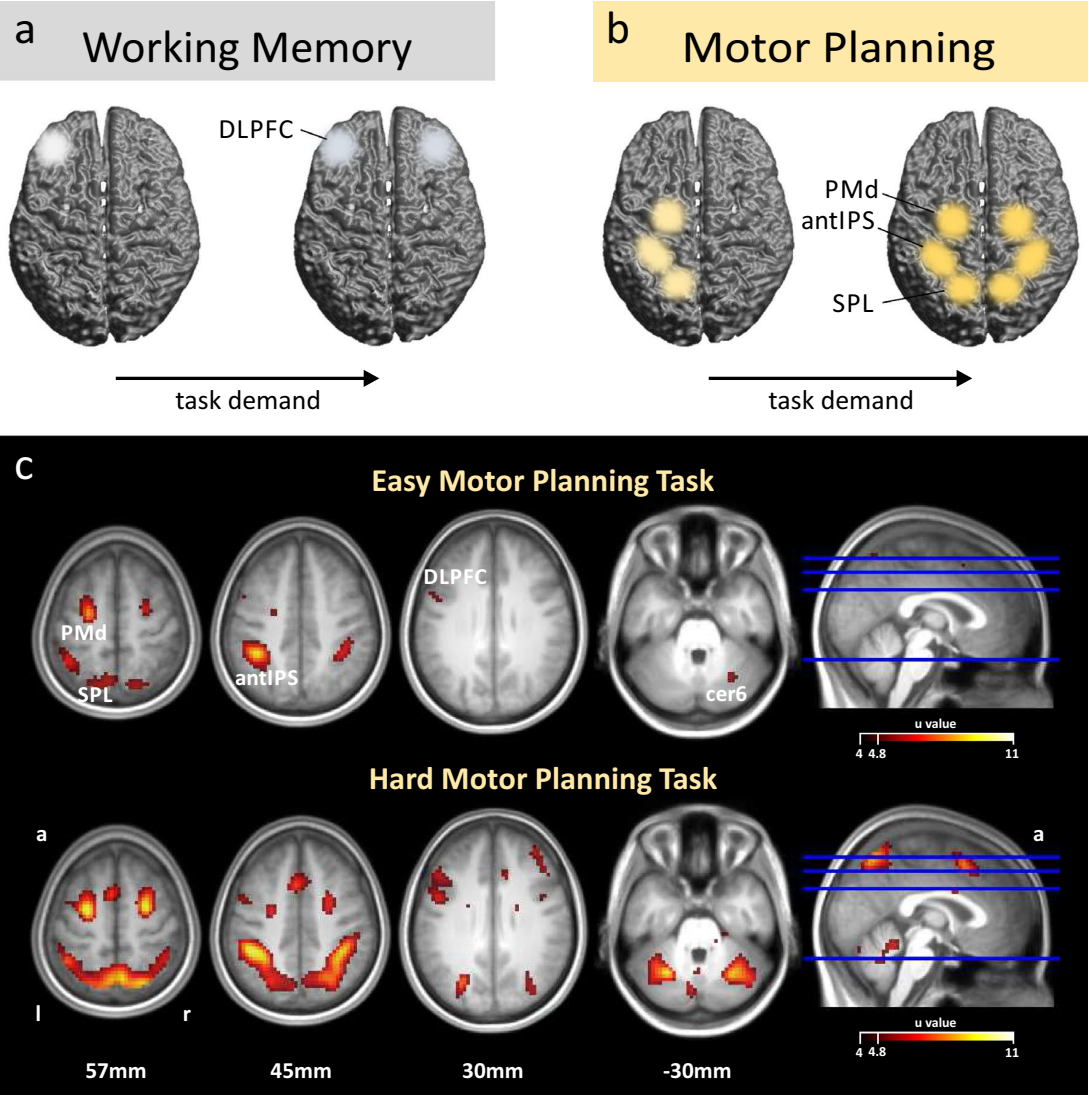
(**a**) Illustration of cross-hemispheric recruitment in young adults. Previously, it has been shown that left-lateralized dorsolateral prefrontal cortex (DLPFC) shows bilateral activation for high processing demands in working memory tasks^24^. (**b**) We hypothesize that such cross-hemispheric recruitment in young adults is present in areas beyond prefrontal cortex and in tasks beyond WM. For our motor planning task we expect respective changes as a function of task demand in otherwise left-lateralized planning regions in the posterior parietal cortex (SPL, antIPS) and premotor cortex (PMd). (**c**) Brain activation across our group of subjects in the action planning study is separately depicted for both the easy condition ‘11’ vs. a control condition (CT) and for the difficult condition ‘4’ vs. CT. Maps are based on maximum pseudo-t permutation statistics (*P* < 0.05 FWE; 5000 permutations; variance smoothed with 7 mm^3^; height threshold: u = 4.81). While a rather unilateral activity pattern surfaces in the easy task condition with stronger activity in left PPC (SPL and antIPS), left PMd, left prefrontal cortex (DLPFC) and the right cerebellum (cer6), this pattern is more bilateral in the hard planning task. l, r, a denote left, right, anterior, respectively.

A recent fMRI study by Höller-Wallscheid and colleagues ^24^ extended these earlier findings in several ways: First, their experiment allowed to directly attribute bilateral activity to increasing load during WM maintenance, as it was temporally isolated from respective activity related to visual stimulus representation and to response execution. This is important, because otherwise changes in lateralization might merely reflect the net of different sub-processes (i.e. stimulus processing, memory maintenance, memory retrieval, response execution, etc.), each of which might exhibit a different pattern of lateralization (compare^25,26^). Second, their study showed that the compensatory cross-hemispheric recruitment in prefrontal cortex does occur across WM domains and, importantly, in any adult independent of age (^24^; also compare^21,27,28^ and see Fig. 1a). Building on this and related research (compare^18,21, 29,30^) we suggest that at least in principle, such an age-independent co-recruitment of the idling hemisphere might assist also other lateralized executive processes beyond working memory and might be present also in brain regions other than prefrontal cortex .

We here focus on such an executive function that - like working memory - also displays pronounced lateralization and in regions beyond frontal cortex, namely action planning^31,32^: For instance, the planning of actions towards spatial objects engages lateralized activity in posterior parietal cortex (PPC), both with respect to the side of the effector and with respect of the visual hemifield in which targets are being presented (see^31^ for a review). In addition, going beyond such effector- and visual hemifield-specific effects, the planning of goal-directed movement seems generally represented more strongly in the left hemisphere - even in left-handers (e.g. see^33^). Also in our own previous research we could show that the planning of goal-directed sequences of movements with the right hand, engages left-lateralized preparatory fMRI-activity in superior parietal lobule (SPL) as well as in dorsal premotor cortex (PMd)^34^. Note that, as was true for the study on retrospective maintenance of WM by Höller-Wallscheid and colleagues mentioned above^24^, our task design ensured that this pattern of lateralization was neither due to visual stimulus representation nor to motor response execution but reflected prospective planning in a delayed response paradigm.

We here hypothesize that increasing the processing demands in such a prospective motor planning paradigm for the right hand could likewise demand a co-recruitment of the idling right hemisphere in young adults and in regions beyond prefrontal cortex such as in PPC or PMd (compare Fig. 1b). Previous research on the co-recruitment of ipsilateral primary motor cortex (M1) during the execution of demanding finger movement sequences^35,36^ seems compatible with such an hypothesis. In fact, the authors of the latter study discuss that the recruitment of ipsilateral M1 might be likely related to upstream planning rather than to movement execution proper (but see^35^ for an opposing view). Accordingly, motor recovery after stroke is associated with increases in activity of contralesional motor cortex and, in addition, with bilateral recruitment of premotor areas (for review see^37^).

To address our hypothesis, we analyze fMRI data from a recent action planning experiment^38^, in which we now focus on hemispheric lateralization as a function of task complexity. We also report corresponding analyses of another set of fMRI data, namely from the aforementioned WM study by Höller-Wallscheid and colleagues^24^. Remember, the study by Höller-Wallscheid and colleagues^24^ demonstrated that, in young adults, fMRI-activity related to the maintenance of verbal and spatial WM material in DLPFC is left-lateralized as long as memory loads are low, while DLPFC activity turned bilateral when WM load and respective task difficulty increased.

Our current analyses of these two data sets goes beyond the previous results in several important ways. First, we can test whether bilateral recruitment due to increasing task demand would also arise in preparatory activity during the motor planning task decribed by Schach et al.^38^, namely in otherwise lateralized brain areas within and beyond prefrontal cortex. Note that the initial description of their experiment focused on a different research question (compare discussion) and did not report activity estimates for both hemispheres. Second, by additionally considering the dataset from Höller-Wallscheid and colleagues^24^, we can contrast any neural substrates engaged in bilateral recruitment in motor planning with those engaged in visual and spatial WM maintenance across tasks and experiments. Crucially, both experiments share the same principle task design: in both cases a delayed response task allowed to temporally separate sustained fMRI-activity during the delay phase from activity related to stimulus presentation and response execution (e.g. compare^34,39^). In other words, the task epoch, which we analyzed across both tasks in terms of lateralized brain activity, was quasi-identical but differed only with respect to the cognitive operations performed, namely the prospective planning of goal-directed actions^38^ versus the maintenance of retrospective information^24^, respectively. Finally, while our principle analyses closely followed the procedures described by Höller-Wallscheid and colleagues^24^, we now chiefly quantified changes in cross-hemispheric recruitment by means of a lateralization index^40,41^. This allowed us detecting any changes of lateralization with increasing difficulty in both datasets. Instead, their original approach focused on *“strict bilaterality”*, namely by testing whether activity in lateralized areas becomes indistinguishable across hemispheres when subjects were operating at their performance limit.

Our measure of lateralization for memory maintenance during the delay confirms that increases in WM load lead to a stronger co-recruitment of DLPFC in the idling right hemisphere (cf.^24^). More importantly, our results further reveal that such cross-hemispheric recruitment with increasing task demand is present also in delay-related planning activity and in areas beyond prefrontal cortex, namely in PMd, in PPC and in the cerebellum. Finally, we demonstrate that cross-hemispheric recruitment with increasing WM load also operates beyond prefrontal cortex: at least for the maintenance of spatial WM do areas in PPC display a significant shift of their lateralization index towards bilaterality with increasing load. Our study suggests that young adults co-recruit task-specific areas in the idling hemisphere whenever a lateralized executive function approaches its capacity limit. It contributes to a better understanding of hemispheric interaction in terms of optimal resource allocation for performing complex cognitive tasks with limited capacities.

## Results

### Prospective action planning

**Figure 2:**
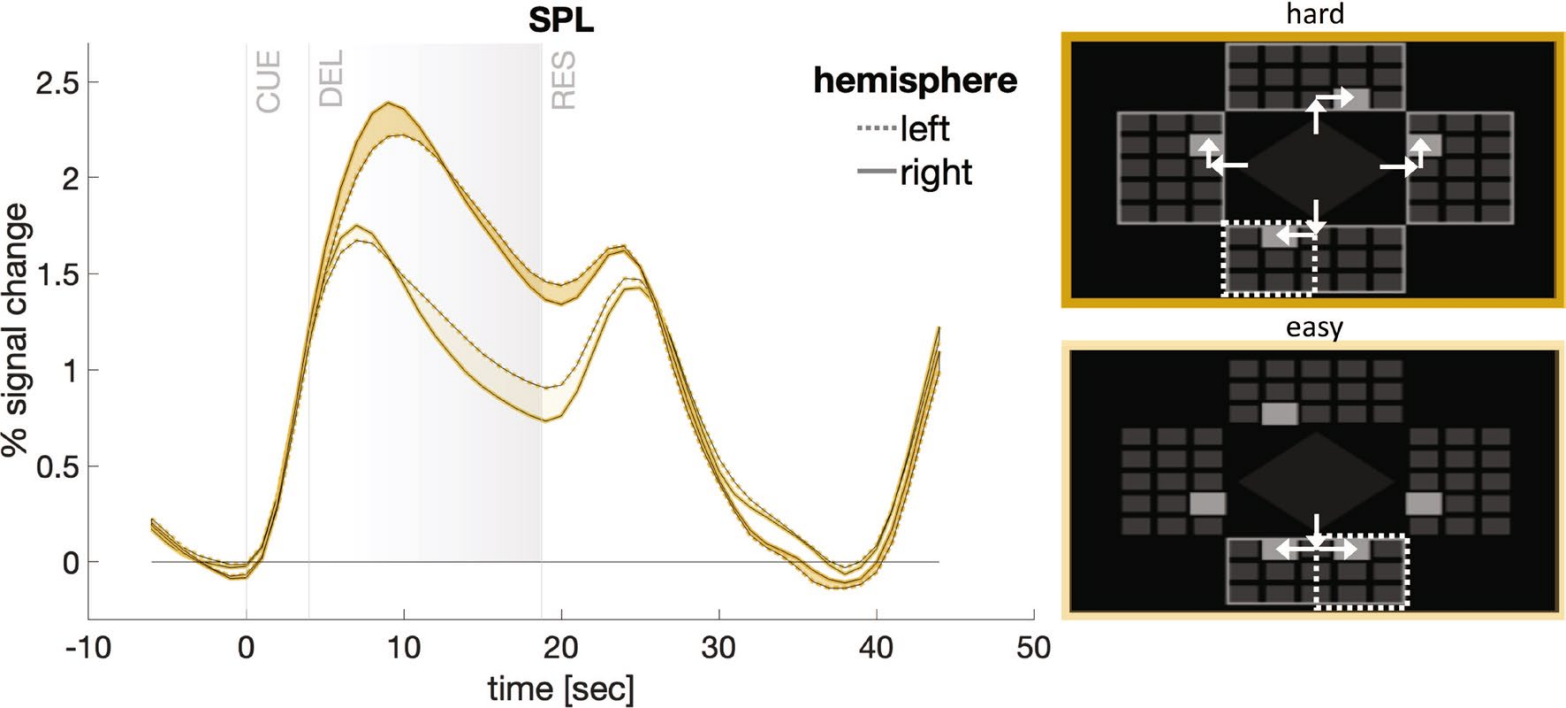
Time course of the fMRI signal changes in left and right SPL as a function of task demand in the motor planning experiment. The actual task is illustrated on the right. In the hard task condition, subjects were instructed to plan four distinct movement sequences (compare arrows for illustration) towards four potential targets (light grey boxes) in different panels (left, right, up, down), whereas in the easy planning condition, only two partially overlapping sequences towards two potential targets in one panel had to be planned. Information about the location of the target relevant for action execution was only provided during the response phase (dotted frame). Increasing task demand led to higher fMRI signal amplitudes in both hemispheres. Importantly, the left hemispheric dominance in sustained planning-related activity in SPL during the late delay phase (DEL; grey shaded area) decreased with increasing task load. Note that during the late delay period, fMRI-activity can neither be accounted for by any preceding stimulus presentation (CUE) nor by movement execution, which is happening later during the response phase (RES). Time courses reflect the mean calculated across 19 subjects and are temporally aligned to stimulus onset (CUE). The beginning of the delay (DEL) and of the response phase (RES) is indicated in addition.

The first fMRI dataset, which we investigated, stemmed from a delayed response task (DRT). Using this task, we had studied concurrent prospective planning of action sequences towards remembered visual targets in a group of young adults^38^. Specifically, in this task an initial “cue” revealed multiple potential target locations (light grey boxes in those target areas highlighted by a light grey frame; compare Fig. 2). During a subsequent delay period with central fixation on an otherwise dark background for 14-16.5 seconds, subjects were instructed to remember all potential targets and to prepare alternative responses to each of them. However, they received information about the one relevant target location only after this delay, namely through an additional “selection” go-signal presented in the response phase (dotted frame in Fig. 2). The actual response then required subjects to guide a visual cursor from the center of the screen towards the relevant target using a respective sequence of up/down/left/right cursor button presses with their right hand (compare Fig. 2). Importantly, planning complexity was varied across four target planning conditions in that a different number of potential target locations was initially cued: subjects were instructed to plan a single movement sequence towards one target (’1’), two partially overlapping sequences towards two potential targets in one panel (’11’; compare “easy” example in Fig. 2), two distinct movement sequences towards two potential targets in different panels (’2’), or four distinct movement sequences towards four potential targets in different panels (’4’ compare “hard” example in Fig. 2), respectively (see methods section “Experiment I: Concurrent Motor Planning” for further details). As our WM data set from Höller-Wallscheid et al. ^24^ only consisted of one easy and one hard condition, we only considered two planning conditions, namely ’11’ (“easy”) and ’4’ (“hard”). We chose these conditions because they required the same cognitive operations (WM, planning, target selection, etc.) but varied in task demand (i.e. memorizing and planning towards 2 vs. 4 potential targets, respectively) and because they led to performance levels that were closest to those in our WM data set, as detailed below (compare Results section “Prospective Action Planning-*Functional hemispheric lateralization*”).

#### Behavioral performance

The behavioral performance analyses showed that subjects’ reaction times and error rates significantly increased with increasing task complexity (two-sided Wilcoxon signed rank test, $p<0.001$, $r=-0.849$ and $p=0.003$, $r=-0.676$). Mean reaction time was 951.9 ± 14.8 ms for easy trials and 1078.2 ± 19.6 ms for difficult trials. The mean error rate was 0.078 ± 0.020 in the easy condition and 0.144 ± 0.022 in the hard condition. Movement times in both planning conditions were not significantly different (easy: 747.6 ± 12.8 ms, hard: 751.9 ± 11.5 ms; two-sided Wilcoxon signed rank $p=0.658$, $r=-0.102$).

Additionally, based on the error rate measure, we computed subjects’ throughput (^24,42^, also compare the formula in the Methods section “Analyzing Subjects’ Performance”). This measure provides an estimate about how many plans (or targets) subjects had been able to form (or maintain) during the delay period. The throughput is calculated from the hit rate performance (hit rate - chance level) as a ratio to the error-free performance (1 - chance level) multiplied by the number of movement plans (items) in each condition. Throughput was on average lower with 1.805 ± 0.176 in the easy (2 targets/plans) condition compared to 3.281 ± 0.479 in the difficult (4 targets/plans) condition (two-sided Wilcoxon sign rank $p<0.005$, $r=-0.885$).

#### Functional hemispheric lateralization

Figure 2 shows an exemplary time course of the raw task-related fMRI signal changes in left and right SPL. This time course illustrates how the delayed response task enabled us to study sustained fMRI activity related to action planning during the delay, which can be clearly separated both from an earlier peak in activity related to cue presentation and a later peak associated with response execution. Note a characteristic increase of the fMRI activity during the delay phase in the hard (’4’) as compared to the easy (’11’) planning condition. A further visual comparison of planning-related activity during the late delay phase in the left- vs. the right-hemispheric counterparts of the SPL shows a left-hemispheric dominance in the easy condition and a more bilateral hemispheric recruitment in the hard condition.

Figure 1c exhibits areas that were commonly activated by the easy and the difficult motor planning task across our group of subjects. During the easy task, a more unilateral activation pattern surfaced, comprising of stronger activity in left PPC (antIPS and SPL), left PMd, left DLPFC and the right cerebellum. As expected (compare Fig. 1b), this activation pattern turned into a more bilateral one for the difficult task condition. Note that while this illustration might provide a first impression about how lateralization patterns during motor planning might change as a function of task difficulty, these maps do not provide a statistical proof for such a change (compare^43^). To investigate lateralization we used the same region of interest- (ROI-) based approach as applied by Höller-Wallscheid et al.^24^. In brief, we identified task-related brain activity during the planning period of our task, independent of task difficulty. This was done separately in each individual to account for inter-subject variability in functional activation loci despite normalization procedures. Regions that exhibited sustained, planning-related activity were frontal areas (DLPFC and PMd), areas in PPC (antIPS, SPL), areas within the cerebellar hemispheres 6 and 8 (cer6 and cer8, respectively) and anterior insular cortex (AIC) (also compare Supplementary Table S1 and Methods section). For each of these bilateral ROIs we calculated a lateralization index (LI)^41^ based on the respective GLM-beta estimates of the delay phase. This was done separately for both task conditions, easy and hard (for details see Methods section). Throughout, the LI values were defined as the relative normalized difference between the signal contribution of left vs. right hemispheres. LI values range from -1 to 1 for completely right-lateralized activation vs. fully left-lateralized activation, respectively. Note that the same ROI-based analytical procedures were conducted for both data sets (Prospective Motor Planning and Retrospective Working Memory). In addition, and to be more directly comparable to the study of Höller-Wallscheid and colleagues^24^, we in addition conducted Bayes factor (BF) analyses to estimate the evidence in favor of unilaterality (H1) vs. strict bilaterality (H0; for details please refer to the Methods section). A BF >3 thereby denotes substantial evidence in favor of H1 (unilaterality), while a BF<1/3 denotes substantial evidence for H0 (bilaterality)^44^.

**Figure 3:**
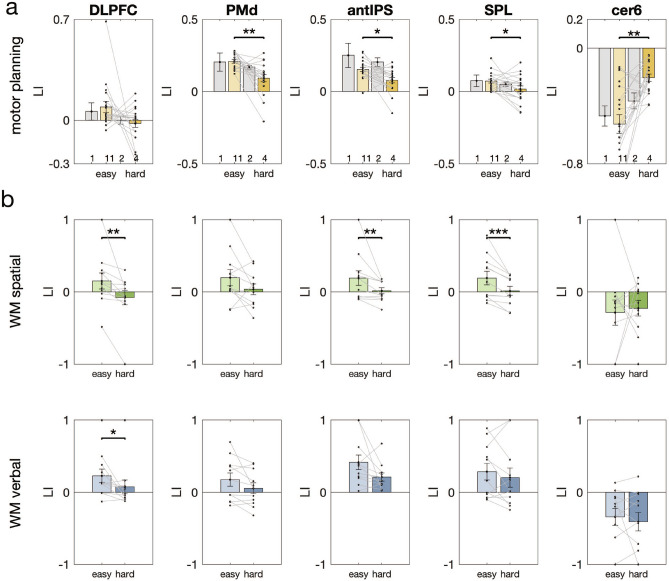
Hemispheric lateralization. (**a**) For the motor planning task, lateralization indices showed significant changes across task conditions with varying load (easy: ’11’ vs. hard: ’4’) in task-related areas. In the premotor cortex and posterior parietal brain areas (PMd, SPL and antIPS), left-hemispheric dominance was reduced with increasing task demand. In the cerebellum (cer6) activity shifted from a right-hemispheric lateralization to bilateral activity, when task demand increased. (**b**) For the spatial and verbal working memory task, lateralization indices decreased significantly in conditions with high (hard) compared to low task-load (easy) in prefrontal brain areas (spatial: DLPFC, SPL and antIPS; verbal: DLPFC [and aPFC, compare Supplementary Fig. S1], two-sided Wilcoxon signed rank test). We indicated statistical differences in LIs as a function of task demand with *** for $p < 0.001$, ** for $0.001<= p < 0.01$, and * for $0.01<= p < 0.05$.

In a first step, we assessed the lateralization of each bilateral ROI in the motor planning task (compare Fig. 3a and Supplementary Fig. S1). This was done by statistically testing whether or not subjects’ LIs in the easy condition (’11’) were different from zero (two-sided Wilcoxon signed rank test, Bonferroni-corrected for multiple comparisons across ROIs). As to be expected for the easy condition (compare Introduction), we found left-lateralized activity in PMd ($p=0.0009$, corrected, $r=0.8871$) and in parietal areas antIPS ($p=0.001$, corrected, $r=0.8678$) and SPL ($p=0.0015$, corrected, $r=0.8494$). Right-lateralized activity was obtained from the cerebellum cer6 ($p=0.0009$, corrected, $r=-0.8774$) and cer8 ($p= 0.0015$, corrected, $r=-0.8494$). DLPFC, which we consider in addition due to its cross-hemispheric recruitment in working memory, exhibited a left-lateralization during easy motor planning ($p=0.0062$, corrected, $r=0.6278$). Correspondingly, the Bayes factor analyses revealed evidence in favor of unilaterality across these ROIs that ranged from substantial to decisive (compare Supplemental Fig. S2). Area AIC did not exhibit any significant lateralization in the easy planning condition but, instead, there was substantial evidence in favor of bilaterality of AIC. This area will therefore not be considered any further.

In a second step, we compared the respective LIs as a function of task demand (two-sided Wilcoxon signed rank test). Figure 3a and Supplementary Fig. S1 show a respective depiction of the LIs as across subjects’ means $\pm ~SE$ as well as LIs of individual subjects for the different task conditions and for all ROIs. As was already mentioned above, we considered the task conditions ’11’ and ’4’ as relevant for our comparison between “easy” and “hard” prospective action planning, as they showed comparable performance across the two studies in terms of the average error rates in the easy task conditions (verbal WM: 0.004%; spatial WM: 0.006%, Motor Planning: 0.078%) as well as in the difficult task conditions (verbal WM: 0.159%; spatial WM: 0.167%, Motor Planning: 0.144%). In accordance with our hypothesis, we observed a significant decrease of LI in PPC (SPL, $p=0.0269$, $r=0.5078$ and antIPS, $p=0.0158$, $r=5539$) and PMd ($p=0.0017$, $r=0.7201$) with increasing task demand (easy: ’11’ vs hard: ’4’), indicating a cross-hemispheric recruitment of the right hemisphere relevant during concurrent prospective motor planning, when task demand increases. A corresponding pattern emerged in the cerebellum (cer6, $p=0.0038$, $r=-0.7478$ and cer8, $p=0.004$, $r=-0.6647$), where the right-lateralization in the easier task condition (’11’) becomes more bilateral when task demand gets higher (’4’). Note that we did not have any prior hypothesis about such cross-hemispheric recruitment in the cerebellum. We therefore would like to stress that the change in lateralization in cer6 and cer8 would also survive Bonferroni-correction for testing multiple ROIs. In DLPFC ($p=0.0836$, $r=0.3970$) we merely reveled a trend for changes of the LI in our motor planning task. Note, however, these changes of lateralization in DLPFC-and in all other planning-ROIs-were significant, when considering larger areas of activation (averaging across a sphere of 9 mm instead of 3 mm; compare Supplementary Table S2). We performed this analysis in an analogous manner to the approach of Höller-Wallscheid and colleagues^24^, namely to account for the possibility that larger areas of activation might contribute to the lateralization pattern. Further following these authors’ approach, we investigated lateralization also in independently defined ROIs. ROIs were now defined by the MNI-coordinates for antIPS, SPL and PMd provided by a meta analysis on action planning^45^. The related analyses of the LIs led to the same qualitative results (Supplementary Table S2). Finally, when defining ROIs based on group activity coordinates derived from an independent planning condition (condition ‘2’) the same qualitative differences in lateralization surfaced across all planning-ROIs (Supplementary Table S2).

Additionally considering the results of the related Bayes-factor analyses, suggests that both DLPFC and SPL thereby represented strict bilaterality in the difficult planning condition, while - as compared to the easy condition - activity in PMd, antIPS and the cerebellum (cer6) was less left-lateralized (compare Supplemental Fig. S2).

#### Performance Benefits as a Function of Hemispheric Lateralization

In an exporatory analysis we asked whether the aforementioned changes in lateralization during action planning would be accompanied by a behavioral benefit, analogous to the behavioral benefit in WM demonstrated by Cabeza et al ^18^. To this end, we split our subject group in three thirds (compare to^46^), namely according to their LI values in the hard condition and separately for DLPFC, SPL, antIPS, PMd, cer6 and cer8. We then tested whether the third of subjects with the strongest cross-hemispheric recruitment for a given ROI would exhibit a smaller increase in RT (with respect to the easy condition) than the third of subjects with the smallest cross-hemispheric recruitment. As depicted in Supplementary Fig. S3, we found such LI-dependent performance benefit for SPL (one-sided Wilcoxon rank sum test, Bonferroni corrected for multiple comparisons across ROIs, $p = 0.038$, $r=0.9935$; note that cer8 exhibited the same principle effect but at $p = 0.0465$ uncorrected, $r=0.3857$, while the results for the remaining ROIs were $p>0.05$ uncorrected).

### Retrospective working memory

The second experiment that we focus on was a delayed-response task involving the maintenance of retrospective information in working memory as a function of task demand. Since this study has been described in detail before^24^, we here chiefly focus on our new LI-based analysis of this dataset. In addition, we replicate earlier results of Bayes-factor analyses. But before reporting the respective results, we first briefly describe the principle design of this study: Two separate working memory domains were tested in this study, namely the verbal and the spatial domain. In each domain two different load levels were tested (easy and hard). To this end subjects were sequentially presented with a different number of verbal/spatial items during an initial encoding phase. In the easy condition 3 verbal or 2 spatial items were presented, respectively, while 7 verbal or 6 spatial items were presented in the difficult condition. During a subsequent delay phase of 15-16 seconds they then had to maintain the presented items in memory (note that the length of the delay was comparable to that of the prospective action planning task). Finally, during a response phase, subjects had to retrieve these items from working memory in order to identify a newly presented target item (see methods section “Experiment II: Working Memory” for further details).

#### Behavioral performance

Subjects’ behavioral performance was measured as the proportion of hits and the throughput computed after^24, 42^ as a measure of items a person is able to successfully keep in memory. In the easy condition hit rate was on average 0.996 ± 0.005 (verbal domain, 3 items) and 0.984 ± 0.008 (spatial domain, 2 items). In the difficult condition the hit rate was significantly lower (two-sided Wilcoxon sign rank $p<0.005$ for both domains) with 0.841 ± 0.036 (verbal domain, $r=0.8474$) and 0.833 ± 0.0039 (spatial domain, $r=0.8457$).

In the easy condition, the throughput was 2.980 ± 0.021 (verbal domain) and 1.938 ± 0.033 (spatial domain). Throughput values in the difficult condition were significantly higher (two-sided Wilcoxon sign rank $p<0.005$ for both domains) with 5.701 ± 0.296 (verbal domain, 7 items, $r= -0.8851$) and 4.797 ± 0.282 (spatial domain, 6 items, $r=-0.8851$)^24^.

#### Functional hemispheric lateralization

We calculated LIs based on the GLM-betas estimates from the delay-phase for those ROIs that exhibited sustained working memory-related activity (compare^24^ for details). ROIs comprised areas in frontal cortex (aPFC, DLPFC, VLPFC, PMv and PMd), PPC (antIPS, SPL), the cerebellum (cer6) and insular cortex (AIC). Note, to allow a qualitative comparison of changes in lateralization across the two data sets on action planning and working memory, we here chiefly focus on those ROIs that could be identified in both data sets (compare Fig. 3; for the remaining ROIs please compare Supplementary Fig. S1). LIs were determined separately for the easy and the hard working memory load condition and separately for the verbal and the spatial working memory task.

Figure 3b shows LIs expressed as group means $\pm ~SE$ and as individual subject data points. For WM maintenance in the verbal task domain, we found a significant difference in LI between the easy and hard task condition in the DLPFC ($p=0.0195$, $r=0.6915$). Also for the spatial task domain we could find significant changes in lateralization in DLPFC ($p=0.003$, $r=0.8310$).

This confirms the results of the study by Höller-Wallscheid et al.^24^, which assessed lateralization using different analytical approaches. Going beyond this earlier study, we found significant changes in lateralization in aPFC ($p=0.0137$, $r=0.7238$) for the verbal memory task (see Supplementary Figure S1). In addition, we revealed changes in lateralization for the spatial memory task in PPC (SPL, $p=0.001$, $r=0.8847$ and antIPS, $p=0.002$, $r=0.8578$). Note that the change in lateralization in SPL and antIPS also survives Bonferroni-correction for testing multiple ROIs in experiment II. In other words, also in ROIs beyond prefrontal cortex, working memory-related fMRI activity shifted from a left hemispheric dominance in easy task conditions to a more bilateral pattern when task demand increased. The corresponding results of the Bayes-factor analyses has been already reported before^24^, with a focus on the emerging bilateral activation pattern in DLPFC during the difficult WM condition in both the spatial as well as the verbal domain and for aPFC in the verbal domain (compare Supplemental Fig. S2). In addition, we’d here like to highlight that the significant changes in the LI in PPC during spatial WM maintenance were associated with substantial to strong evidence in favor of bilaterality in the difficult task condition (Supplemental Fig. S2).

### Comparing functional hemispheric lateralization across tasks and experiments

In a final set of analyses we compared LIs as a function of difficulty across tasks and experiments. These analyses were restricted to those 5 ROIs reported by both experiments. Note that in all cases these ROIs had been functionally identified in slightly different ways across tasks and in different subjects across experiments. ROIs do thus slightly vary in their location (compare Supplemental Table S1) but still reside within the same gross anatomical structure. For our analyses we performed a repeated-measures ANOVA to compare the LIs as a functions of the within-subject factors task (spatial vs. verbal WM) and condition (easy vs. difficult) in the retrospective working memory study. We additionally compared LIs across experiments by means of two mixed-model ANOVAs with the within-subject factor condition (easy vs. difficult) and the between-subject factor task (motor planning vs. spatial WM and motor planning vs. verbal WM, respectively). To account for multiple statistical comparisons (N=3), we Bonferroni-adjusted the critical p-value to 0.016. For a complete depiction of the related results (please refer to Supplemental Table S3). In the retrospective memory study we did not reveal any significant influence of task or an interaction of task and condition. Yet, there was a significant influence of condition (i.e. difficulty) in DLPFC ($p=0.011$). An impact of condition on the LIs in DLPFC was likewise present when comparing motor planning vs. spatial WM (p=0.004; task and task x condition: n.s.). The same influence was present also for PMd, antIPS, SPL, and cer6. Only SPL thereby exhibited an additional task x condition interaction ($p=0.012$), namely due to a more pronounced laterality in the easy spatial WM condition. The factor task was not significant in all cases. Finally, when comparing the motor planning vs. the verbal WM task, we again revealed a significant impact of condition in DLPFC ($p=0.008$; task and task x condition: n.s.) as well as in PMd (p<0.001; task and task x condition: n.s.). The antIPS and the cerebellum (cer6) also exhibited a significant influence of the factor condition ($p=0.007$ and $p=0.006$, respectively), while the earlier was accompanied by a significant task effect ($p<0.001$) and the latter by a significant task x condition interaction ($p=0.004$): This was because in antIPS left lateralization was generally much more pronounced in the verbal WM task and because in cer6 a modulation of the LI with difficulty was present only in the motor planning task but not in the verbal WM task.

## Discussion

While various previous studies have investigated cross-hemispheric recruitment of executive functions in the elderly (e.g. see^18,29,30^), only few studies have looked at bilateral activation in young adults, mostly with a focus on lateralized areas in prefrontal cortex in WM tasks^24,30,47^. Our question was, whether we could find evidence for cross-hemispheric recruitment beyond prefrontal cortex across different executive tasks and in various brain regions as a general information processing strategy. Consequently, we have investigated how changes in hemispheric lateralization arise from a task-dependent adjustment of brain resources in young adults.

What distinguishes our approach from various related studies is the fact that our analyses allowed us to “zoom in” on well-defined task-related ROIs as well as on specific executive processes (retrospective working memory and prospective action planning). It thereby follows the suggestion to study the lateralization of functionally and anatomically circumscribed sub-processes rather than to focus on overall task-related activity or on the hemispheres as a whole^26^. Specifically, all experiments were designed as delayed response tasks, which allowed to focus on fMRI-activity related merely to cognitive processing (here prospective action planning and retrospective memory maintenance) during the delay phases in absence of activity of sensory coding or of action execution which per se do lateralize and might vary as a function of task demand. The similarity of our experiments (e.g. comparable delay times) and the use of the same analysis methods on all data sets thereby enabled us to compare results across tasks and experiments. Note, however, the direct quantitative comparison between tasks is difficult to interpret, because of the differences across tasks with respect to functional ROI definition (compare above) and with respect to performance, particularly in the easy conditions. Another feature of our work relates to the fact that we looked at various task-related brain regions that were functionally defined in each individual. We took this approach to account for differences in functional activation loci across individuals despite brain-normalization procedures. Note that we carefully considered the criteria defined by Kriegeskorte and colleagues^48^ to avoid circularity. Crucially, we obtained comparable qualitative results also for ROI coordinates that were independently defined on the group level or for coordinates from meta-studies on WM and action planning, respectively (compare Supplementary Tables S2 in^24^ and in this publication). In the following, we will discuss our results in detail.

Figure 1 summarizes the hypothesized load-dependent changes in hemispheric lateralization for both tasks, which we ultimately validate by computing LIs from left and right hemispheres in different task-related ROIs. In line with these hypotheses, our results could identify cross-hemispheric recruitment during high task demand in brain areas beyond prefrontal cortex and also in the prospective action planning task. Importantly, greater task demand was thereby accompanied by a significant increase of subjects’ level of executive processing, as was assessed by our throughput measure. This is compatible with the idea that, at least in our study, cross-hemispheric recruitment is not unspecific but could actually further performance. With increasing prospective motor planning demand we observed a change from left-lateralization to more bilateral hemispheric activation beyond prefrontal cortex, namely in PPC (antIPS and SPL) and PMd. We also observed planning-load induced cross-hemispheric recruitment in the left hemisphere of the otherwise right-lateralized cerebellum (cer6 and cer8). While these results will be further discussed in detail below, we now first focus on the results of our WM task.

In the retrospective WM tasks, our analysis methods confirmed significant changes in DLPFC lateralization during the maintenance of verbal and spatial items with different memory load (compare Höller-Wallscheid et al.^24^). Our results further revealed that increasing memory load during spatial WM stimulates a co-recruitment of posterior parietal areas (antIPS and SPL) in the right hemisphere. These parietal areas have been previously shown to contribute to spatial WM (e.g. compare the meta-analysis by Wager  & Smith 2003 ^49^ and Rottschy et. al. 2012^50^). Likewise, in our own previous research we obtained evidence for a contribution of these areas to both spatial memory and action planning^34,38^. Interestingly, a recent MEG study by Proskovec and colleagues^51^ further revealed a stronger beta-desynchronization (and thus activation of) right SPL during spatial WM maintenance that was associated with better performance. This result complements our findings and is consistent with the idea that a co-recruitment of the “other” hemisphere could support WM performance also in areas beyond prefrontal cortex. Note that a putative limitation of our results on WM-related changes in lateralization might be the comparatively small sample size in experiment II. We discussed this issue already at length in the original paper of Höller-Wallscheid (please see the discussion section in ^24^). Importantly, by additionally analyzing data across tasks and experiments, we obtained further support for these “bilaterality”-effects.

In the action-planning task, we could show cross-hemispheric recruitment in areas that have already previously been shown to contribute to the prospective planning of goal-directed movements, namely in posterior parietal cortex (SPL and antIPS) and in premotor cortex (PMd)^34^. Our results fit to our expectation of bilateral recruitment in these otherwise lateralized movement planning ROIs, that might be evoked by an increase of the required task-specific processing power when approaching the capacity limit of a single hemisphere. For our movement planning task - and only for this task - we additionally revealed cross-hemispheric recruitment of the otherwise right-lateralized cerebellum. As the cerebellum is well-known for its role in motor control and coordination^52^, the measured activity changes in of lobule VI and VIII of the cerebellum fit well to the idea of a task-specific co-recruitment of the other hemisphere. In fact, an fMRI study by Haaland et al. ^53^ also showed a greater bilateral contribution of the cerebellum during the execution of more complex movement sequences. In summary, the changes in hemispheric lateralization as a function of task demand in our motor task clearly suggest that a co-recruitment of the other hemisphere in young adults is present also during prospective planning and in areas beyond prefrontal cortex.

One obvious question related to the latter results is whether the cross-hemispheric recruitment in PPC, PMd and the cerebellum could be explained by increased retrospective working memory load rather than prospective planning. This is because in our action planning experiment, the concurrent prospective planning of actions clearly required a memory about the target locations, too. Moreover, working memory activity has been described for all of the aforementioned areas (e.g.^24,34,49,50,54^). Hence, does the measured activity indeed represent the information processing for planning movements even in the hard condition or, alternatively, would subjects select an alternative “memory strategy” when approaching their capacity limit for concurrent planning? The latter strategy would only entail memorizing the information of the potential targets during the delay and postpone the planning to the response period, in which the ultimate target is identified. In our previous study on the same dataset^38^, we specifically addressed this question in a model-based analysis and could demonstrate, that fMRI-signal modulations in all respective areas are significantly better explained by model predictions that consider a concurrent prospective planning strategy compared to a serial decision-making pipeline, where the delay phase would only consist of retrospective memory activity. From this we conclude, that delayed-response fMRI activity in our task (at least partially) consists of planning-related activity and not only reflects memory maintenance. More importantly, when combining the results of this earlier study with our current findings, we can show that the increase of these model-estimates of planning activity as a function of task difficulty (rather than the related estimates for retrospective memory) significantly predict changes in lateralization in PPC, PMd and the cerebellum (compare Supplemental Table S4).

Compatible with the idea that the aforementioned changes in lateralization related to prospective planning rather than to retrospective memory, we further demonstrate that subjects with a more bilateral activation pattern in SPL also exhibited the type of reaction time benefit that commonly accompanies successful planning (compare^38,55^). This result is consistent with a paramount role of SPL in the planning of visually guided movement^34^. Future work should probe whether such behavioral benefit in action planning through cross-hemispheric recruitment can be mediated by areas beyond SPL (such as PMd or the cerebellum). Yet, as experiment I was not designed to address this interrelation, further studies with larger samples are certainly needed. Finally, it would be also interesting to see whether such planning benefit through cross-hemispheric recruitment would surface in older adults too.

Indirect support for performance benefits through cross-hemispheric recruitment in various task-domains also comes from other tasks, for example from visual field studies. Such studies often report that the distribution of visual stimuli across both visual hemi-fields (as compared to feeding only a single hemisphere through within hemi-field presentation) leads to an increase in subjects’ performance. This effect has been termed “bilateral processing advantage” (BPA)^56^ and was observed in different task settings such as perceptual matching^57,58^, mental rotation^59^ or visual WM^60,61^. The aforementioned behavioral effects could plausibly correspond to a compensatory recruitment of bilateral areas in various task domains lending credence to the notion of a general information processing principle.

Going beyond these visual field studies and the results of our work, there is some evidence for bilateral recruitment as a function of task demand also from tasks other than WM and for brain areas beyond frontal cortex. As was already pointed out in the introduction, the increased recruitment of ipsilateral M1 with increasing difficulty may serve as another example for the motor domain (e.g.^35,36^). Yet, the detailed mechanisms underlying this effect are still somewhat unclear: is it due to a coordinating role of ipisilateral M1 during movement execution or rather due to increased planning, potentially even mediated by upstream motor planning areas such as SPL and PMd (also compare^62^)? Despite M1 was not considered a primary ROI, a post-hoc analysis of its activity in our action planning study revealed that it also displayed a significant shift in lateralization during the delay-period with increasing task difficulty, namely from left-lateralization to strict bilaterality (compare Supplementary Figs. S1 and S2). This is at least in agreement with the idea that ipsilateral M1, PPC and PMd are jointly recruited during the planning of difficult tasks. Studies demonstrating a contribution of these areas to motor recovery after stroke are also compatible with this latter view. This is true despite the fact that the residual recruitment of ispilesional M1 so far seems the major determinant for patients’ recovery on the longer run (for review see^37^). Similarly, one recent study did demonstrate an age-dependent HAROLD effect for M1 but elderly did not benefit from the effect^63^. Examples of stroke patients with left-lateralized brain damage with functional recovery from speech or semantic processing deficits likewise demonstrate a cross-hemispheric recruitment in the right hemisphere when compensating prior loss of these functions (e.g. see^64,65^). Finally, we’d like to highlight recent research in healthy subjects in which increasing levels of uncertainty also led to bilateral activation of otherwise left-lateralized prefrontal activity during a task-switching paradigm^66^.

A mechanism that could account for bilateral recruitment in several of the aforementioned cases (including our own research) could constitute a division of labor between hemispheres in terms of another executive process common to both, namely attentional processing^56^. Hence, the overall increase in performance could be attributed to increased attention and vigilance with higher task difficulty rather than to a dynamic resource allocation for specific task-related processing^67,68^. Indeed, right-lateralized activity in various cerebro-cortical areas is attributed to attention and vigilance^7,69^ and, accordingly, a related increase in activity in our right hemispheric ROIs due to higher attentional task demands might have led to the more bilateral activation pattern in these otherwise left-lateralized regions. A similar interpretation has been put forward also in the task-switching study by Tsumura and colleagues^66^ mentioned above. However, increasing levels of vigilance seemingly lead to bilaterality itself - namely through a co-recruitment of the left hemisphere^70^. Moreover, our experimental investigations revealed that different tasks have evoked bilateral recruitment only in task-specific areas such as in the cerebellum for planning but not for verbal WM. This casts doubt on the notion that changes in inter-hemispheric activation only reflect attention and not more specific aspects of task processing that require extended resources due to higher cognitive load. We suggest that a compensation for increasing task demand should as well recruit task-specific mechanisms depending on distinct brain circuits.

The concrete mechanisms underlying cross-hemispheric recruitment in difficult tasks needs to be clarified in future work. For example, we need to further investigate how cross-hemispheric interaction relates to an adjustable distribution of processing resources and how information transfer between hemispheres is managed across the corpus callosum^71^. For instance, it was shown that DLPFC, as a domain-general region, exhibits a stronger cross-hemispheric coupling for high task demand than earlier “perceptual areas” (ventral temporal regions as domain-specific regions), which instead do display a decrease in their functional connectivity between hemispheres^57^. This finding is in agreement with our interpretation, namely that the mechanism of interhemispheric activation seems to be specific with respect to both the functional processes and brain regions being involved. Also in our work, bilaterality in DLPFC could reflect a domain general role in executive processing across tasks and experiments (compare Supplemental Table S3; also see Höller-Wallscheid et al.^24^ for further discussion). At the other end, cross-hemispheric recruitment in the cerebellum could reflect a domain-specific role in motor planning.

Finally, we’d like to discuss whether cross-hemispheric recruitment due to increasing task demand leads to less lateralization, strict bilaterality, or even to a reversed lateralization. The combination of our LI measure with the BF-analyses (contrasting laterality vs. strict bilaterality) at least allowed us to describe the respective patterns of lateralization in our tasks on the group level. In all reported cases, we always observed less lateralization in the difficult condition. For DLPFC, the lesser lateralization always led to strict bilaterality. In case of the spatial WM task, antIPS and SPL also exhibited strict bilaterality in the difficult condition. The same was true also for PMd. Note however, that this area only exhibited a change in lateralization in our across-task analyses. Finally, like DLPFC, did SPL exhibit strict bilaterality for the motor planning task. We never observed a reversed lateralization pattern. In this respect, our results are fully compatible with the idea of a hemispheric asymmetry reduction, as postulated by the HAROLD model^18^, though this effect was obviously present in younger adults, too. The latter might derive from the fact that (different to^18^) our subjects were operating at their performance limit (compare^24,38^). Interestingly, despite subjects worked at their limit, the asymmetry reduction only led to strict bilaterality across all tasks in a single ROI, namely DLPFC. Hence, strict bilaterality perhaps reflects a special case, that surfaces only in some tasks and brain regions. We’d like to add that the overall pattern of our results is well compatible with the so-called CRUNCH model (compensation-related utilization of neural circuits hypothesis^27^). This model provides a suitable explanation for the neural changes dependent on subjective task demand while going beyond cross-hemispheric recruitment. Within the framework of this model bilateral recruitment could present a special case of a compensatory mechanism for an overextending task that exhausts the individual capacity of a neural resources^24,72^. In other words, compensatory processes other than cross-hemispheric recruitment could likewise be triggered by increasing task demand and maybe even be better captured by CRUNCH^73^.

In summary, our work suggests that cross-hemispheric recruitment of executive processing during high task demand is not limited to retrospective working memory and to prefrontal cortex. Such recruitment is also at work during prospective planning and in other brain regions, namely in dorsal premotor cortex, posterior parietal cortex and in the cerebellum. We hope that our preliminary findings will stimulate future research that will directly test the notion that the co-recruitment of the idling hemisphere serves as a general strategy of the lateralized brain to cope with limited capacities of executive processes across different brain regions and tasks and to optimize performance.

## Methods

We performed a secondary analysis on two data sets that both recorded fMRI during task performance with different task-load conditions. The first data set was collected by Schach et al. ^38^ and comprises fMRI recordings during performance of a motor planning task with varying complexity in terms of prospective parallel planning of multiple potential movement paths. The second data set was a control experiment collected by Höller-Wallscheid et al. ^24^ in a group of young subjects and comprises a spatial and verbal working memory task with varying numbers of visual items to memorize. Both previous studies were approved by the ethics committee of the Faculty of Medicine at the University of Tübingen (837/2019BO2 and 365/2010BO2) and all methods were carried out in accordance with the relevant guidelines and regulations. For both studies, all participants provided informed consent.

### Experiment I: prospective action planning

#### Subjects and experimental paradigm

In the motor planning study (experiment I) by Schach et al. ^38^, data of nineteen healthy subjects (11 females, 8 males; mean age: 27.5; SD: 4.5) were recorded and included in the analysis. All subjects reported to be right-handed.

They performed a delayed-response task (DRT) with varying planning complexity to prepare sequential finger movements. Each trial started with an initial fixation period of 13.5–16.0 seconds, which served as an implicit baseline for fMRI.

Task complexity in the different target planning conditions was defined by the number of potential targets distributed over four panels that were shown to the subjects in the cue phase of each trial (compare Fig. 2). The cue phase lasted 3 seconds, followed by a 1 second visual mask to prevent afterimages of the cue. Subjects were instructed to concurrently prepare movement plans to all relevant targets shown during the delay phase. The duration of the delay was chosen randomly between 14–16.5 seconds. The original study contained four target planning conditions ( $c_T \in \{$’1’, ’11’, ’2’, ’4’$\}$) in which subjects had to consider 1, 2 or 4 targets, distributed across 4 target zones (panels). The relevant panel(s) were thereby indicated in each individual trial. The 2-fold planning conditions ’11’ and ’2’ thereby varied in the “regional proximity” – either both targets were in the same target panel (’11’; compare the “easy” condition in Fig. 2) or distributed over two (out of four) different panels (’2’). The experiment in addition differentiated three possible target distances: targets could be reached through either 2, 3 or 4 steps. During the final response phase (3 seconds), subjects then used a button-box operated with their right hand to stepwise guide a visual cursor from the center of the screen to the memorized position of the relevant target. The relevant target was thereby only indicated during the response phase, namely through a visual frame around the relevant target area (compare Fig. 2). The experiment was recorded in four consecutive blocks consisting of 30 trials each (one subject had five blocks, two subjects only three blocks) with 6 trial repetitions of each of the four target planning condition and 6 additional trials of a control condition without planning (CT). Conditions were presented randomly interleaved. We here only focus on one easy (’11’) and one hard (’4’) condition, pooled across the different step conditions, to make our results better comparable to those of experiment II, which only comprised 2 conditions (also compare above). For full transparency, we still depict respective data for conditions ’1’ and ’2’ as well (compare Fig. 2).

#### fMRI data acquisition

MRI images were acquired on a 3T Siemens PRISMA scanner and with a 20-channel headcoil (Siemens, Ellwangen, Germany) using a gradient-echo planar (EPI) sequence (48 sclices, 64x64 voxel resolution, 3x3 mm inplane voxel size, slice thickness=3 mm, gap=0 mm, repetition time=2000 ms, echo time=35 ms, field of view=192x192 mm, flip angle=75 deg) for functional $T2^*$-weighted volumes. Overall we collected 2400 EPIs per subject (600EPIs collected across 4 individual runs). For two subjects, who prematurely terminated the recordings, we obtained 1800 and 2177 EPIs, respectively. One subject performed a 5th run (3000 EPIs).

### Experiment II: retrospective working memory

#### Subjects and experimental task

 In the working memory study conducted by Höller-Wallscheid et al. ^24^ as a control experiment, 11 healthy subjects (5 females, 6 males; mean age: 25.1; SD: 3.2) were included in the analysis. Subjects were right-handed according to the Edinburgh Handedness Inventory^74^.

In the delayed match-to-sample task, each trial started with an initial fixation period of 15–16 seconds, which served as an implicit baseline. This period was followed by the sequential presentation of memory items (1 second each plus a 0.2 second inter item interval). These items had to be maintained in working memory throughout a subsequent delay period of 15–16 seconds. During a 10 seconds response phase subjects then had to verbally identify one item that had been changed. The experimental conditions covered two different load levels of verbal (different consonants) and spatial (different targets on a rectangular grid with 20 cells) visual stimulus material. The easy condition consisted of 3 items in the verbal domain and 2 items in the spatial domain. The difficult condition consisted of 7 items in the verbal domain and 6 items in the spatial domain. The experiment was recorded in four sessions with 20 trials each. Each session covered trial repetitions of a single experimental condition, beginning with the easy conditions of both task domains (verbal easy and spatial easy, session 1 and 2, randomized between subjects) followed by the hard conditions of both task domains (verbal hard and spatial hard, session 3 and 4, randomized between subjects).

#### fMRI data acquisition

MRI images were acquired on the same 3T Siemens PRISMA scanner and with a 20-channel headcoil. Imaging parameters varied slightly: We used a gradient-echo planar (EPI) sequence (32 sclices, 64x64 voxel resolution, 3x3 mm inplane voxel size, slice thickness=3.2 mm, gap=0.8 mm, repetition time=2000 ms, echo time=35 ms, field of view=192x192 mm , flip angle=90 deg) for functional $T2^*$-weighted volumes. We collected 460 and 470 EPIs in the easy spatial and verbal WM condition while in the difficult conditions we collected 520 and 530 EPIs for spatial and verbal WM, respectively.

### Analyses of both experiments

#### fMRI analyses

A similar analysis approach was chosen for both data sets. Data from both correct and incorrect trials entered our analyses in all cases. Functional image processing, including preprocessing, individual level (first-level) analysis and group level (second-level) analysis was conducted with SPM8 in case of the WM study^24^ and with SPM12 for the action planning study^38^ (SPM, Wellcome Centre for Human Neuroimaging, London, UK). Note, we here did not redo these fMRI analyses, namely to allow that our additional measures of cross-hemispheric activity can still be directly compared to the results presented in our original studies. In the following we briefly describe the basic fMRI analyses applied in our earlier works.

The preprocessing covered spatial realignment of all functional images to the first EPI image as a reference, co-registration of the mean functional EPI image with an anatomical T1 image, spatial normalization to the Montreal Neurological Institute space (MNI-template) with voxel size of 3 × 3 × 3 mm^3^ for EPI images and spatial smoothing of the normalized EPIs with a Gaussian kernel (7 mm full-width at half maximum (FWHM) Gaussian filter (please find details in^24,38^).

In a subject-specific analysis first-level, a general linear model (GLM) was specified. The GLM of experiment I comprised 35 regressors, 5 for all task conditions (4 target planning conditions ’1’, ’11’, ’2’, ’4’ + control condition) during the cue phase (CUE) and (5x3)x2 for all task conditions and all step conditions (2-4 steps) during the planning delay (DEL) and response phase (RES). The GLM of experiment II covered 3x2 model regressors (betas) for the three trial phases (encoding, maintenance, response) and both task load conditions (easy, hard). In all cases regressors were defined by the onset and duration of each respective task epoch and modelled by the haemodynamic response function of SPM. The parameters of the motion correction procedure were included as regressors of no interest.

We defined regions of interest (ROI) for individual subjects (see Supplementary Table S1), from which we extracted the beta estimates of the planning delay phase of 14-16.5 sec (experiment I) and the maintenance phase of 15-16 sec (experiment II). Further details on the ROI selection and analysis can be found in the next section (Regions of interest analysis) and the method section of the original articles^24,38^. From a 3 mm radius sphere around each individual ROI coordinate we extracted the normalized GLM model mean beta weights of the regressors of interest. Beta estimates for each single ROI consisted of means calculated across 7 voxels in total. To investigate whether our results would be affected by the spatial spread of activity, we repeated our main analyses on mean beta estimates extracted for 9 mm spheres.

#### Region of interest analysis

Task-relevant brain areas were determined based on the group statistics of the second-level analysis on the volumes of each experimental data set. A detailed description on the ROI selection can be found in the corresponding method description of the main papers^24,38^. We applied the following approach that was common in both studies: First, in a second-level analysis of experiment I, we exhibited significant activities in all brain areas that are typically involved in motor planning or visual memory maintenance ([Delay of ’1’, ’11’, ’2’, ’4’] > CT). The group contrast of experiment II mapped activity related to working-memory maintenance (Delay > baseline). We identified five brain areas, that were involved in both tasks and that exhibited activity in both hemispheres. Those areas were dorsal premotor cortex (PMd), posterior parietal cortex (superior parietal lobule (SPL) and anterior intraparietal sulcus (antIPS, in^24^ referred to as IPS), dorsolateral prefrontal cortex (DLPFC), and lobule VI of the cerebellum (cer6). Additional task-relevant ROIs were considered for analysis of experiment I (lobule VIII of the cerebellum (cer8)) and for the analysis of experiment II (anterior prefrontal cortex (aPFC), ventrolateral prefrontal cortex (VLPFC), and ventral premotor cortex (PMv)). Moreover, for experiment I we additionally included primary motor cortex (M1) as a control ROI. M1 was defined by the statistical contrast (Response > baseline). In each individual subject, we then used the respective group ROI coordinates in order to map her individual ROI coordinates to best account for inter-subject anatomical variability in functionally defined brain regions. This was done by identifying the coordinates of the local statistical maximum that was within the same anatomical structure and closest to the group coordinate in the subject specific t-contrast (experiment I: [Delay of ’1’, ’11’, ’2’, ’4’] > CT; experiment II: Delay > baseline). For the motivation of this procedure please also refer to the paper by Höller-Wallscheid and colleagues^24^, which also describes the ROI assessment for experiment II in great detail. Also in experiment I we followed the same principle approach ^38^ but, in addition, considered hemispheric symmetry in the loci of activation as an additional selection criterion for a bilateral ROI. For the cerebellar hemispheres we had to relax our statistical threshold criteria in 3 subjects ($p<0.1$ uncorrected)) and defined the corresponding cross-hemispheric counterparts in the cerebellum by mirroring the respective coordinates to the other hemisphere.

In addition to this individual subject-based ROI-localization approach, we also used coordinates from an earlier meta-study^45^ to allow repeating our main analyses for the action planning study. We had applied the same approach in our study on WM already before^24^. In addition, we also calculated a group contrast for [Delay of’2’] > CT (using procedures, which will be described below under Group Activation Maps) to now define ROIs across subjects and through a condition that was not further analyzed (note that our statistical comparisons of measures of lateralization considered conditions ‘11’ and ‘4’, only). The results of these additional analyses are presented in Supplementary Table S2.

#### Analyzing ROI-specific fMRI lateralization

To quantify the hemispheric dominance as a function of task conditions, we calculated lateralization indices (LI) in the predefined ROIs. For the analysis of experiment I, we used the GLM-beta estimates of the planning delay period (DEL) in the easy and hard target planning conditions ’11’ and ’4’, where we averaged over the three “step conditions”. In the same way, we analyzed the GLM-beta estimates of the maintenance phase of experiment II in the two task conditions and for the easy and hard variants of these tasks, respectively.

We determined the fMRI-based LI values using a variant that also allows for negative quantities^41^. LI values are computed with $\displaystyle LI = \frac{Q_{LH}-Q_{RH}}{|Q_{LH}|+|Q_{RH}|}$ as the relative normalized difference between the contribution of left ($Q_{LH}$) and right ($Q_{RH}$) hemispheres according to^40^, quantifying hemispheric dominance.

We additionally calculated Bayes factors (BFs) for every ROI on the acquired beta-estimates according to Dienes^75^. These factors quantified how probable the alternative hypothesis (H1: there are differences in ROI activity between the hemispheres) is versus the null hypothesis (H0: there are no differences in ROI activity between the hemispheres). We modeled the prediction of our alternative hypothesis as a uniform distribution. In every ROI, we determined which of the two hemispheric counterparts exhibited the higher beta estimate and used this as an upper limit of the model and we chose a value representing 5 percent of this value as the lower limit.

#### Group activation maps

To illustrate activation across our group of subjects in the action planning study for both the easy condition ‘11’ vs. CT as well as for the difficult condition ‘4’ vs. CT, we performed a group analysis using the respective contrast images from individuals. These contrast images were analyzed using permutation statistics using the SnPM13-toolbox for SPM (p<0.05 FWE; 5000 permutations; maximum pseudo-t statistic; variance smoothed with 7 mm^3^). Note that the same procedure was also applied to determine group-based ROI coordinates for the contrast between ‘2’ vs. CT (compare Region of Interest Analysis).

#### Analyzing subjects’ performance

Subjects’ performance is expressed by means of reaction times, error/hit rates and throughput (for details compare^24,38^). Throughput was calculated according to the formula provided in^24,42^: $\displaystyle \text {Throughput} = \frac{(\text {hit rate} - \text {chance level})}{(1 - \text {chance level}) } \cdot \text {n}$, with n as the number of movement plans/items.

#### Statistics and reproducibility

We performed statistical analysis using SPSS (version 26; IB; SPSS Statistics) and Statistics and Machine Learning Toolbox (Matlab Version 2021a, Mathworks). To test subjects’ behavioral performance in the easy compared to the difficult task conditions, we computed two-sided Wilcoxon signed rank tests. To examine which ROIs showed lateralized planning activity during experiment I, we first calculated two-sided Wilcoxon signed rank test of LI during the delay phase in the easy task condition compared to zero (equal to zero: bilateral, unequal to zero: unilateral). The significance level was adjusted according to the Bonferroni’s procedure, namely by multiplying p-values by the number of ROIs considered in experiment 1 (i.e. 7). We tested for differences between LI during the delay phase between the different levels of task-demand both in experiment I (’11’ as an easy condition vs. ’4’ as the most difficult condition) and in experiments II (easy vs. hard) using two-sided Wilcoxon signed rank tests. Effect sizes ($r=$z-score/*√n*, $n=$number of total observations) were calculated after Rosenthal ^76^. Note that we repeated our analyses using permutation tests (means across subjects; 5000 permutations). These tests led to the same qualitative results as the Wilcoxon signed rank tests and, therefore, will not be reported here.

### Supplementary Information

Supplementary Information.

## Data Availability

The analysis is based on data from two previously published peer-reviewed studies. All relevant data are available under 10.18725/OPARU-50281.
