# Supplementary material for: Cross-hemispheric recruitment during action planning with increasing task demand
Source: Sci Rep. 2023 Sep 16;13:15375. doi: 10.1038/s41598-023-41926-4 (PMC10505196; doi:10.1038/s41598-023-41926-4)
Supplement: Supplementary file 1 — Supplementary Information. [file 41598_2023_41926_MOESM1_ESM.pdf]

Cross-hemispheric recruitment during action planning with increasing task demand

Sonja Schach[1,*], Daniel Alexander Braun[1], and Axel Lindner[2,3,*]

[1]Institute of Neural Information Processing, University of Ulm, Ulm, Germany
[2]Tübingen Center for Mental Health, Department of Psychiatry and Psychotherapy, University of Tübingen, Tübingen, Germany
[3]Centre of Neurology, Division of Neuropsychology, Hertie-Institute for Clinical Brain Research, University of Tübingen, Tübingen, Germany
[*]sonja.schach@uni-ulm.de, a.lindner@uni-tuebingen.de

Table S1. Individual ROI coordinates in MNI space in mm averaged over subjects (±SD) for the experimental tasks.

	Experiment I: Concurrent Motor Planning						Experiment II: Working Memory spatial						Experiment II: Working Memory verbal					
	x	±	y	±	z	±	x	±	y	±	z	±	x	±	y	±	z	±
DLPFCl	−38	6	30	4	28	7	−37	5	34	8	33	7	−39	4	32	8	31	6
DLPFCr	37	6	35	5	30	7	41	6	34	6	31	6	40	4	34	8	33	8
PMdl	−22	11	−6	4	57	6	−29	4	−8	4	63	6	−30	5	−3	9	58	4
PMdr	25	3	−3	4	55	6	29	3	−5	3	60	6	28	5	−3	5	60	6
antIPSl	−36	5	−44	5	45	7	−40	7	−47	6	51	6	−34	8	−51	7	46	7
antIPSr	36	5	−41	5	45	7	41	3	−46	8	51	7	37	5	−50	7	46	7
SPLl	−16	5	−66	3	58	6	−16	5	−68	6	60	5	−17	6	−69	6	55	5
SPLr	18	5	−66	4	58	6	18	6	−68	4	58	5	16	7	−68	3	57	4
cer6l	−29	5	−58	8	−30	4	−29	3	−64	3	−29	3	−30	1	−57	5	−30	3
cer6r	32	6	−58	7	−30	3	35	7	−65	4	−29	3	30	4	−61	4	−29	3
AICl	−33	5	20	5	5	4	−35	4	17	5	5	4	−35	4	19	6	5	5
AICr	35	5	20	5	4	5	36	3	20	5	6	4	36	4	17	6	7	3
cer8l	−32	6	−62	7	−53	4												
cer8r	32	5	−63	6	−53	4												
M1l	−37	3	−21	4	60	4												
M1r	35	9	−17	12	57	8												
aPFCl							−35	4	51	5	12	5	−34	4	50	4	14	4
aPFCr							36	4	50	6	14	6	35	6	53	4	13	7
VLPFCl							−52	6	4	4	29	6	−50	7	7	6	26	8
VLPFCr							51	6	7	3	30	5	46	5	11	2	28	6
PMvl							−48	4	−6	4	47	5	−51	5	−7	7	46	5
PMvr							47	6	0	5	46	5	54	4	−3	8	47	3

Table S2. Comparison of main effect in lateralization based on task-demand during prospective motor planning for different ROI coordinate criteria. The observed significant decrease of LI in DLPFC, SPL, antIPS, PMd and cer6 with increasing task demand (easy: '11' vs hard: '4') indicates cross-hemispheric recruitment of the right (for cer6 the left) hemisphere during concurrent prospective motor planning, when task demand increases. The statistical tests for the comparison of "easy" versus "hard" prospective motor planning were robust in all ROIs also when extracting beta estimates from larger regions (9 mm spheres) and also for alternative ROI coordinates defined according to the group contrast '2' $>$ CT or to a meta-analysis (Blangero et. al[45]; their areas PMd, aIPS=antIPS, pIPS=SPL). We indicated statistical differences in LI as a function of task demand with *** for $p < 0.001$, ** for $0.001 <= p < 0.01$, and * for $0.01 <= p < 0.05$. The respective ROI coordinates of our group contrast were -36 -42 39 and 36 -42 42 [mm x/y/z] for left and right antIPS, -6 66 60 and 6 -63 60 for left and right SPL, -24 -9 54 and 24 -3 57 for left and right PMd, -30 -57 -33 (mirrored coordinates) and 30 -57 -33 for left and right cer6, and -42 33 33 (mirrored coordinates) and 42 33 33 for left and right DLPFC.

	DLPFC	PMd	antIPS	SPL	cer6
Subject-individual ROI (9mm)	*	**	**	*	***
Group contrast ROI (9mm)	**	***	***	**	**
Meta-Analysis ROI^{+} (9mm)		***	***	***	

Table S3. Between-Task comparison. For our comparisons we performed a repeated-measures ANOVA to compare the LIs as a functions of the within-subject factors task (spatial vs. verbal WM) and condition (easy vs. difficult) in the retrospective working memory study [A]. We additionally compared LIs across experiments by means of two mixed-model ANOVAs with the within-subject factor condition (easy vs. difficult) and the between-subject factor task (motor planning vs. spatial WM [B] and motor planning vs. verbal WM [C], respectively). To account for multiple statistical comparisons (N=3), we Bonferroni-adjusted the critical p-value to 0.016. The table provides the respective p-values for the factors task, condition (cond) and their interaction (task*cond).

		DLPFC	PMd	antIPS	SPL	cer6
A	p_{task}	0.397	0.986	0.055	0.246	0.121
	p_{cond}	0.011	0.051	0.038	0.03	0.98
	$p_{task*cond}$	0.366	0.633	0.752	0.29	0.525
B	p_{task}	0.993	0.61	0.837	0.338	0.251
	p_{cond}	0.004	0.001	0.001	0.000	0.012
	$p_{task*cond}$	0.272	0.565	0.127	0.012	0.144
C	p_{task}	0.110	0.571	0.000	0.033	0.930
	p_{cond}	0.008	0.000	0.007	0.064	0.006
	$p_{task*cond}$	0.668	0.956	0.144	0.71	0.004

Table S4. LI across different task conditions with varying demand correlate with predicted theoretical information costs for concurrent prospective planning of actions $E[I_2]$ in dorsal premotor (PMd) and posterior parietal cortex (antIPS and SPL) and the cerebellum. Individual correlation coefficients ρ were, on average, significantly different from 0 across the group of subjects. The additional costs to be incurred for memory of target locations $E[I_1]$ were not correlated with changes in LI. Both theoretical information costs $E[I_1]$ and $E[I_2]$ were previously predicted by a bounded rational decision making model applied to the beta estimates for left cerebro-cortical ROIs and for the right cerebellum (cer6) during the delay period of our action planning task (see Schach et. al 2022 for details[38]). We indicated statistical differences in LI as a function of the respective information cost with *** for $p < 0.001$, ** for $0.001 <= p < 0.01$, * for $0.01 <= p < 0.05$, and ns for non-significant results ($p > 0.05$).

	DLPFC		PMd		antIPS		SPL		cer6	
	ρ	$\pm SE$	ρ	$\pm SE$	ρ	$\pm SE$	ρ	$\pm SE$	ρ	$\pm SE$
$E[I_1]$	0.038	0.163	-0.013	0.191	-0.177	0.144	-0.106	0.175	0.18	0.135
$E[I_2]$	-0.009	0.146	*** -0.524	0.141	*** -0.522	0.12	* -0.347	0.152	** 0.387	0.103

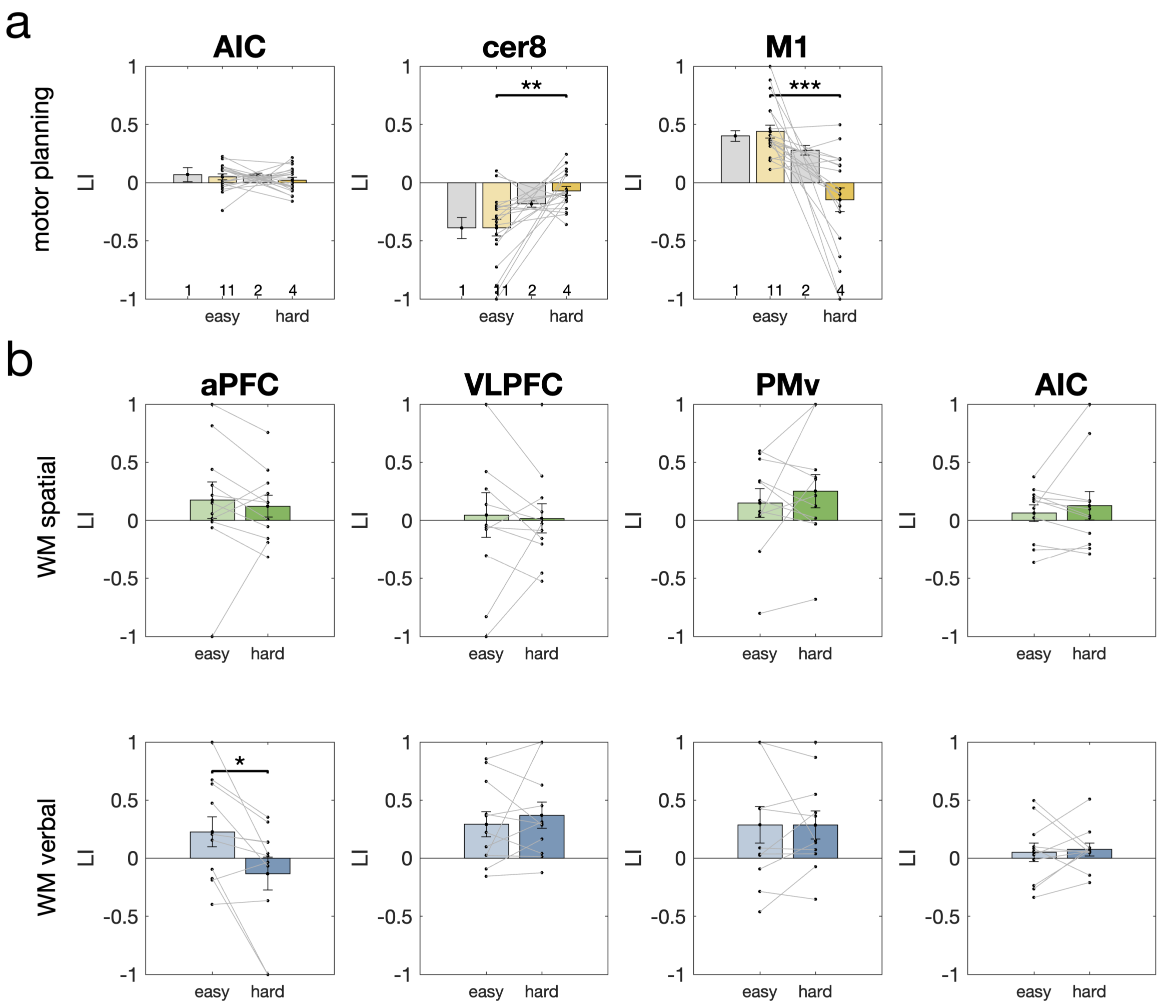

Supplementary Figure S1. Hemispheric lateralization in additional ROIs. **a** For the motor planning task, in the anterior insular cortex (AIC) no significant difference of the LI across task conditions of different complexity was found. In lobule VIII of the cerebellum (cer8), task demand had a significant effect on the LI ($p = 0.0038$, $r = -0.6647$, two-sided Wilcoxon signed rank test). Right-lateralized activation in the easy condition ('11') changed to a more bilateral activation in the hard condition ('4'). A significant effect of task demand is also found for primary motor cortex (M1) ($p < 0.001$, $r = 0.8032$, two-sided Wilcoxon signed rank test). **b** For the verbal working memory task, in the anterior prefrontal cortex (aPFC) the LI was significantly lower in the "hard" condition with high verbal WM task-load compared to the "easy" condition ($p = 0.0137$, $r = 0.7238$, two-sided Wilcoxon signed rank test). In ventrolateral prefrontal cortex (VLPFC), ventral premotor cortex (PMv) and anterior insular cortex (AIC, in Höller-Wallscheid et al. referred to as Insula), no significant effect was found. We indicated statistical differences in LI as a function of task demand with *** for $p < 0.001$, ** for $0.001 <= p < 0.01$, and * for $0.01 <= p < 0.05$.

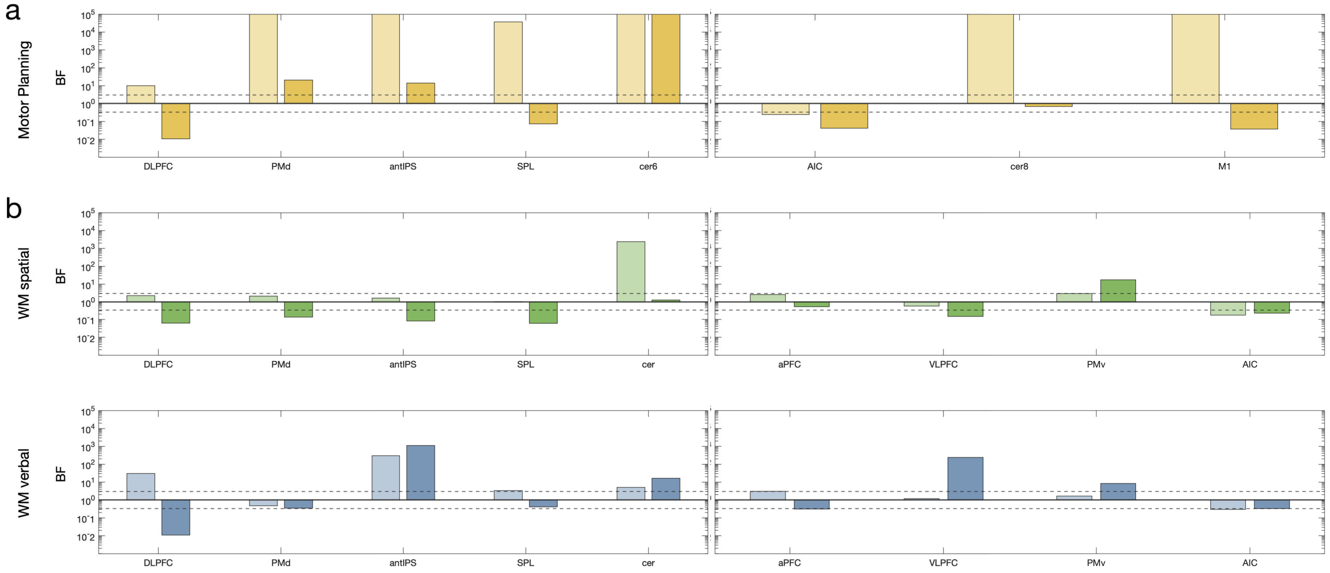

Supplementary Figure S2. Bayes Factor Analyses for the the motor planning experiment (a) and the WM experiment (b) in individual ROIs. The Bayes factor (BF) for the group of subjects is used to distinguish the hypothesis of unilaterality (H1) or bilaterality (H0) in the different task conditions 'easy' (pale colored) against 'hard' (brightly colored). According to Jeffreys[44] substantial evidence for H1 is obtained for $BF > 3$ (upper dashed line) and for H0 in case of $BF < 1/3$ (lower dashed line).

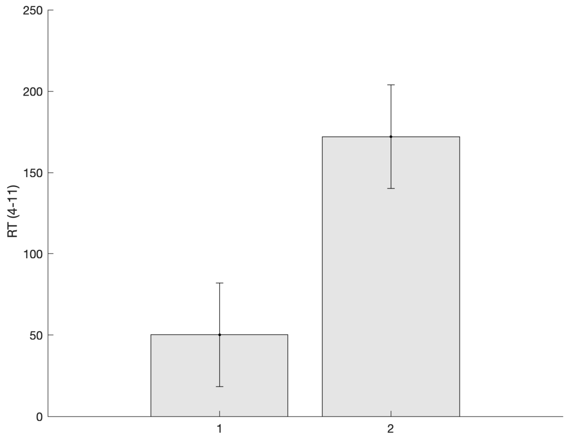

Supplementary Figure S3. LI-dependent performance benefit. The subject group split according to subjects' LI values in the hard condition revealed a behavioral benefit. The third of subjects with the strongest cross-hemispheric recruitment (Group 1) for SPL showed a lower drop in RT for the hard as compared to the easy condition ('4'-'11') as compared to the third of subjects with the smallest cross-hemispheric recruitment (Group 2) (one-sided Wilcoxon rank sum test, Bonferroni corrected for multiple comparisons across ROIs, $p = 0.038$, $r = -0.4421$).
